# Understanding and predicting sensory crispness of deep‐fried battered and breaded coatings

**DOI:** 10.1111/jtxs.12456

**Published:** 2019-07-07

**Authors:** Kha Yiu Voong, Abigail Norton‐Welch, Thomas B. Mills, Ian T. Norton

**Affiliations:** ^1^ School of Chemical Engineering School of Chemical Engineering, University of Birmingham Edgbaston Birmingham UK

**Keywords:** batter, breadcrumbs, crispness, instrumental measurements, sensory evaluation

## Abstract

Crusted crispness refers to coatings with a dry and brittle surface contrasting a high‐moisture core; it is desirable for the enjoyment and quality of deep‐fried goods. This study aims to investigate instrumental measurements and sensory measurements of crispness. Deep‐fried breadcrumb coatings of eight sizes were investigated: 4.0 mm, 2.8 mm, 2.0 mm, 1.4 mm, 1.0 mm, 710 μm, 500 μm, and 355 μm. Sensory profiling was carried out to develop a tailored lexicon for deep‐fried battered and breaded shrimp. Principal component analysis highlights that large breadcrumb sizes correlate with crispness, hardness, particle size, surface color, color uniformity, surface irregularity, total porosity, maximum force, area, drop in force, number of sound peaks, and sound pressure level. Agglomerative hierarchical clustering was used to confirm clustering of samples according to breadcrumb size. Multiple factor analysis confirmed overall correlation between sensory measurements and instrumental measurements (*RV* = 0.810). Partial least squares regression was used to develop a predictive model for crispness from instrumental measurements (*R* ^2^ = .854). The use of texture analysis and Acoustics provide information of the structures strength and deformation behavior, while X‐ray microCT provides a high resolution and noninvasive method that acquires information on the internal morphology. These instrumental methods collectively demonstrate the relationship between microstructure to sensory. This study investigates how a change in the microstructure of deep‐fried battered and breaded coatings affect crispness perception. These changes were investigated analytically and by using a sensory panel, this is important from a manufacturing perspective in order to understand what the major contributors are to a crisp texture. The key highlights of this study include both instrumental measurements and sensory measurements can be used to measure crispness as both types of testing are correlated. Changes in the size of breadcrumbs affect both instrumental measurements and sensory measurements. A predictive model can be re‐simulated to allow prediction of crispness in deep‐fried battered and breaded coatings.

## 1 INTRODUCTION

Deep‐fat frying is a popular method of food preparation that is essentially a heat and mass transfer process. In deep‐fried battered and breaded goods, crispness is a textural parameter that can be used to assess quality and freshness. The perception of crispness is dependent on rheological and mechanical characteristics as well as the sensations during eating (Roudaut, Dacremont, Pàmies, Colas, & Le Meste, 2002). Deep‐fried battered and breaded products are favored by consumers due to their increased palatability provided by a crisp porous outer coating contrasting a tender and high‐moisture core (Antonova, Mallikarjunan, & Duncan, 2003). As well as improving texture and taste, the use of deep‐fried coatings improves appearance and shelf‐life. The loss of crispness is typically due to adsorption of moisture from the atmosphere or water mass transfer from the internal components (Piazza, Gigli, & Ballabio, 2007). In the research described here, the deep‐fried batter and breaded coating of a shrimp product has been studied to understand the major contributors to crispness perception.

### 1.1 Crispness perception

Microstructurally, the arrangement of the structure, composition of constituents, chemical bonds and imperfections will affect crispness perception (Chakra, Allaf, & Jemai, 1996). “Dry” crisp refers to cellular structures with air‐filled cavities surrounded by brittle material that fractures upon being compressed beyond a threshold. The perception of crispness is due to fracturing of this brittle material (Duizer, 2001). Examples of dry crisp include roasted almonds or potato chips (Labuza et al., 2004; Tomasco, 2007). “Wet” crisp refers to cellular structures with turgor, caused by liquid within the cells pressing against the cell walls whilst being oppressed by elasticity and strength of the cell wall. The perception of wet crisp is due to the rupture of these cell walls, therefore increased turgidity increases crispness perception (Duizer, 2001). Examples of wet crisp include fruits or vegetables (Chauvin, Younce, Ross, & Swanson, 2008; Tomasco, 2007). Batter and breadcrumb coatings have properties of both wet and dry crisps and so can be referred to as “crusted” crisp (Tomasco, 2007). The maximum perception of crispness is during the first bite of the mastication process. This is due to the continuous breakdown of the food structure caused by incisors and the hydration of the sample from saliva, thus reducing crispness (Luyten, Plijter, & Van Vliet, 2004).

### 1.2 Instrumental crispness

Characterization of crispness using instrumental measurements have been previously investigated in hopes to try and predict its perception using a combination of morphological characteristics (Adedeji, Liu, & Ngadi, 2011), ultrasonic properties (Antonova et al., 2003), acoustics (Chakra et al., 1996) and force‐deformation measurements (Salvador, Varela, Sanz, & Fiszman, 2009). The type of test or probe must be clearly established, as crispness varies from product to product. The textural term ‘crisp’ has a versatile definition and studies have been carried out on biscuits (Arimi, Duggan, O'sullivan, Lyng, & O'riordan, 2010), nuts (Saklar, Ungan, & Katnas, 1999), breads (Primo‐Martin et al., 2006), and potato chips (Bouaziz et al., 2016) all of which are low‐moisture crisp products. Studies on instrumental crispness of deep‐fried crusted crisp products with a high‐moisture core are limited.

### 1.3 Sensory crispness

In food texture studies, sensory assessment with a trained panel can be used to validate instrumental measurements (Fillion & Kilcast, 2002). The use of descriptive profiling is recognized as an efficient tool for characterizing crispness of fried products (Antonova, Mallikarjunan, & Duncan, 2004; Du Pont, Kirby, & Smith, 1992; Miele, Di Monaco, Formisano, Masi, & Cavella, 2018; Torrico et al., 2019). Crispness of deep‐fried coatings is a versatile textural characteristic that will vary with batter formulation, breadcrumb formulation, frying time and frying temperature. Previous studies have shown that varying breadcrumb size affects the physical and mechanical properties of deep‐fried battered and breaded coatings (Maskat & Kerr, 2002). The optimum time for assessment of crispness can be considered to be during the first bite, this is when surface rupture occurs and moisture has not yet migrated towards the crust (Luyten et al., 2004).

Although instrumental measurements have shown differences in structural parameters between coatings with variable breadcrumb size, sensory assessment is required to identify and distinguish any mouthfeel differences in crispness perception. Studies on high‐moisture crisp products are limited, therefore this study aims to investigate (a) whether a panel is able to visually differentiate between crisp samples, (b) whether a panel is able to identify and describe differences in crispness between samples, (c) investigate any correlations between instrumental and sensory parameters, and (d) develop a predictive model for crispness. By correlating instrumental measurements to sensory data, this will provide useful information for food manufacturers to develop products with a desired level of crispness.

## 2 MATERIALS AND METHODS

### 2.1 Sample preparation

Samples of white shrimp were coated in predust flour, liquid batter layer and breadcrumbs prior to deep‐fat frying. Eight breadcrumb coating sizes were investigated and were separated using the following sieve apertures: 4.0 mm, 2.8 mm, 2.0 mm, 1.4 mm, 1.0 mm, 710 μm, 500 μm and 355 μm. All frozen samples were kept at 8°C before use, then deep‐fried for 3 min at 190°C and kept warm under a heat lamp for an additional 5 min prior to sensory evaluation.

### 2.2 Sensory measurements

A panel of eight was recruited externally according to a set criterion (aged 24–60, six females, two males). A total of three 3 hr training sessions were held in order to familiarize panelists with the sensory attributes (Table 1), followed by one extra 1 hr practice session. The references used for training were set according to Meilgaard et al. (2016). Each attribute was assessed on the first bite and first chew. Spectrum training was employed for both appearance and texture modalities and references were provided according to (Kemp, Hollowood, & Hort, 2009; Meilgaard et al., 2016). Panelists assessed a nine shrimps per day (three different breadcrumb sizes done in triplicate) plus a reference shrimp twice a day. Panelists evaluated one sample at a time at 15 min intervals using a 0–15 Spectrum scale.

**Table 1:** Definitions for sensory attributes used for battered and breaded coatings (Meilgaard, Civille, & Carr, 2016)

	Sensory attributes	Definition	Reference	
Texture	Crispness	The force (noise) with which a product breaks or fractures, characterized by many, small breaks.	Granola bar, club cracker, graham cracker, cheerios, corn flakes, melba toast
	Cohesiveness	The amount of which sample deforms rather than crumbles, cracks or breaks.	Corn muffin, hard breadsticks, cheese, pretzel
	Denseness	The compactness of the sample cross‐section.	Hotdog, malted milk balls, fruit jellies
	Hardness	The force to compress between molars.	Hotdog, peanuts, almonds
Appearance	Crumb coverage	The amount of crumb that fully covers the shrimp product.	0–100% coverage
	Surface uniformity/ irregularity	Lack of smoothness when viewed from a predefined distance.	Smooth vanilla wafer, rough chunky cookie
	Particle size	The size of particles on the sample.	Corn starch 1.0, cornmeal 4.0, regular bread crumbs 6.0, whole wheat bread crumbs 8.0, panko breadcrumbs 9.0, rice krispies 15.0
	Surface color uniformity	Coating color evenness on the surface of the product.	Top of macaroon, vanilla wafer, honey crisp apple, ginger snap
	Surface color	Surface coating color from light to dark.	Top of macaroon, bottom of macaroon

All samples were identified with a random 3‐digit code, then presented so that each panelist received all samples three times in a random order.

### 2.3 Statistical analysis

An analysis of variance (One‐way ANOVA) with post‐hoc Tukey honestly significant difference (HSD) was performed to identify any significant differences between samples. Principal component analysis (PCA) was performed to visualize any relationships among attributes and samples.

Multiple factor analysis (MFA) is a factorial method that has been shown to be useful for studying relationships between different sets of variables, different sets of samples and variables to samples (Ting et al., 2015). An advantage of MFA, is the ability to study both qualitative and quantitative data, but also remove any influences from the sets of variables. Therefore, different sets of variables (i.e., sensory and instrumental data) hold the same weight and influence in the analysis. Agglomerative hierarchical clustering (AHC) analysis was carried out in order to identify clusters within the sample set.

Partial least squares (PLS) regression was performed to study the regression of attributes in order to predict crispness. All statistical analyses were carried out using XLSTAT statistical software 2018.3 (Addinsoft, Paris, France). Model selection function was also incorporated to allow for appropriate variable selection, in order to determine the best model fit. Multicollinearity was confirmed with variance inflation factors and tolerance values, therefore PLS regression was the appropriate method to apply. PLS regression was employed to understand which variables correlated positively or negatively to crispness. It is a recommended analysis if there are a high number of explanatory variables and high correlation between variables (Abdi, 2003; Meilgaard et al., 2016).

### 2.4 Instrumental data

Results from a previous study focused on characterizing eight samples instrumentally. The internal structure of the deep‐fried coatings was evaluated to show how the internal morphology changes with breadcrumb size, as well as the physical and mechanical properties (Voong, Norton, Mills, & Norton, 2018). X‐ray microCT was used to quantify total porosity. Texture analysis and an acoustic envelope were used to quantify maximum compression force, area, sound pressure level, and sound peaks. Changes in porosity, pore size, and structural thickness will subsequently affect the strength of the structure, the rate of oil and moisture transfer, the fracture behavior, and acoustic events.

## 3 RESULTS AND DISCUSSION

### 3.1 Sensory assessment of texture and appearance variables

The mean scores, *p*‐values and significant differences for all texture and appearance attributes are shown in Tables 2 and 3. In terms of appearance attributes, as breadcrumb size decreases from 4.0 mm to 355 μm, “surface color,” “color uniformity,” “particle size,” and “surface irregularity” all decrease significantly (*p*‐value <.0001). The only significant increase is “crumb coverage” (*p*‐value .002). Increasing crumb coverage can be explained as breadcrumb size decreases, crumbs become more uniform in shape and size, this allows strong adhesion to the batter due to packing ability and therefore, surface coverage and surface uniformity (Fiszman, 2008). A smoother surface with highly packed particles will result in fewer protrusions exposed during frying. Therefore, this explains low surface irregularity, lightness in color and color uniformity.

**Table 2:** Mean scores and *p*‐values for appearance attributes of deep‐fried battered and breaded shrimp of varying breadcrumb size

	Surface color	Color uniformity	Particle size	Surface uniformity/irregularity	Crumb coverage
4.0 mm	7.67^a^	8.23^a^	10.0^a^	9.23^a^	14.1^c^
2.8 mm	7.73^a^	8.10^a^	9.44^a^	8.81^ab^	14.1^c^
2.0 mm	7.31^a^	7.42^b^	8.50^b^	8.21^bc^	14.3^bc^
1.4 mm	6.81^b^	6.58^c^	7.33^c^	7.50^c^	14.4^abc^
1.0 mm	6.60^c^	6.06^cd^	6.38^d^	6.46^d^	14.7^a^
710 μm	6.79^b^	6.08^c^	6.08^d^	6.42^de^	14.5^ab^
500 μm	5.77^c^	5.35^e^	4.92^e^	5.50^ef^	14.6^ab^
355 μm	5.75^c^	5.40^de^	4.40^e^	5.38^f^	14.5^ab^
*p*‐value	<.001	<.001	<.001	<.001	<.001

*Note*: Different letters in the same column refer to a significant difference (*p* < .05) according to Tukey's HSD.

**Table 3:** Mean scores for texture attributes of deep‐fried battered and breaded shrimp of varying breadcrumb size

	Crispness	Hardness	Cohesiveness	Denseness
4.0 mm	9.10^a^	9.06^a^	4.13^bc^	7.21^a^
2.8 mm	8.90^ab^	8.75^ab^	3.85^c^	7.33^a^
2.0 mm	8.02^bc^	8.38^b^	3.94^bc^	7.40^ab^
1.4 mm	8.13^bc^	8.42^b^	4.33^abc^	7.73^abc^
1.0 mm	6.96^de^	7.71^c^	4.44^abc^	7.65^abc^
710 μm	7.38^cd^	7.77^c^	4.52^ab^	8.21^c^
500 μm	6.67^de^	7.73^c^	4.83^a^	8.08^c^
355 μm	6.25^e^	7.35^c^	4.83^a^	7.98^bc^
*p*‐value	<.001	<.001	<.001	<.001

*Note*: Different letters in the same column refer to a significant difference (*p* < .05) according to Tukey's HSD.

Larger breadcrumbs are amorphous in shape and have large porous protrusions, which are exposed during frying oil and will therefore darken in color during frying.

In terms of texture attributes, as breadcrumb size decreases from 4.0 mm to 355 μm, “crispness” decreases significantly (*p*‐value <.0001) and “hardness” decreases significantly (*p*‐value <.0001). Alternatively, “cohesiveness” increases significantly (*p*‐value .029), “denseness” also increases significantly (*p*‐value .015). As stated previously, larger breadcrumbs have higher exposure to frying oil. Therefore, crispness and hardness is expected to be higher as moisture and oil exchange is prolonged. This drying process results in a brittle coating with low moisture. Denseness and cohesiveness have a higher mean score for smaller breadcrumb sizes. This can be explained as increased packing ability allows particles to firmly stick to the batter, this results in less crumbling and cracking.

### 3.2 Correlation of variables

**Figure 1:**
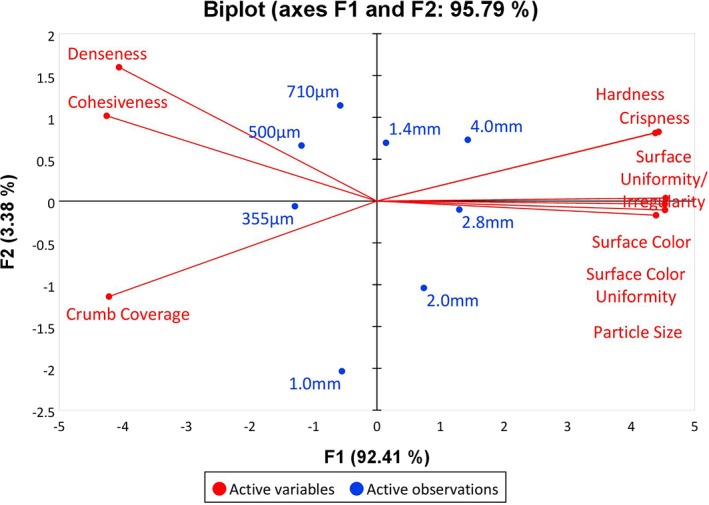
PCA biplot of first two principal components explaining 95.79% of sensory texture and appearance attributes. Observations refer to samples of deep‐fried battered and breaded coatings with varying breadcrumb size. PCA, principal component analysis

Correlation PCA was used to study the overall structure of the dataset to allow a visual understanding of relationships between variables, between samples and between variables to samples. PC1 and PC2 described 92.41 and 3.38% of the data variability with an eigenvalue of 8.317 and 0.304. Therefore, the first two components were sufficient enough for interpreting both appearance and texture sensory data as they accounted for a total of 95.79% of the variation, respectively (Figure 1). The sensory variables that best describe the appearance and texture variability in the samples are heavily loaded onto the first principal component as this component has a high number of attributes with high positive loadings. Hardness, crispness, surface irregularity, surface color, surface uniformity and particle size are positively correlated to PC1, whilst crumb coverage, cohesiveness and denseness are negatively correlated to PC1. Sample 4.0 and 2.8 mm are close in proximity, suggesting similarities that are characterized by hardness, crispness, surface irregularity, surface color, color uniformity and particle size.

Sample 355, 500, and 710 μm are close in proximity, suggesting similarities characterized by crumb coverage, denseness and cohesiveness. Samples 2.0, 1.4, and 1.0 mm do not appear to be in close proximity to any other sample or attribute.

**Figure 2:**
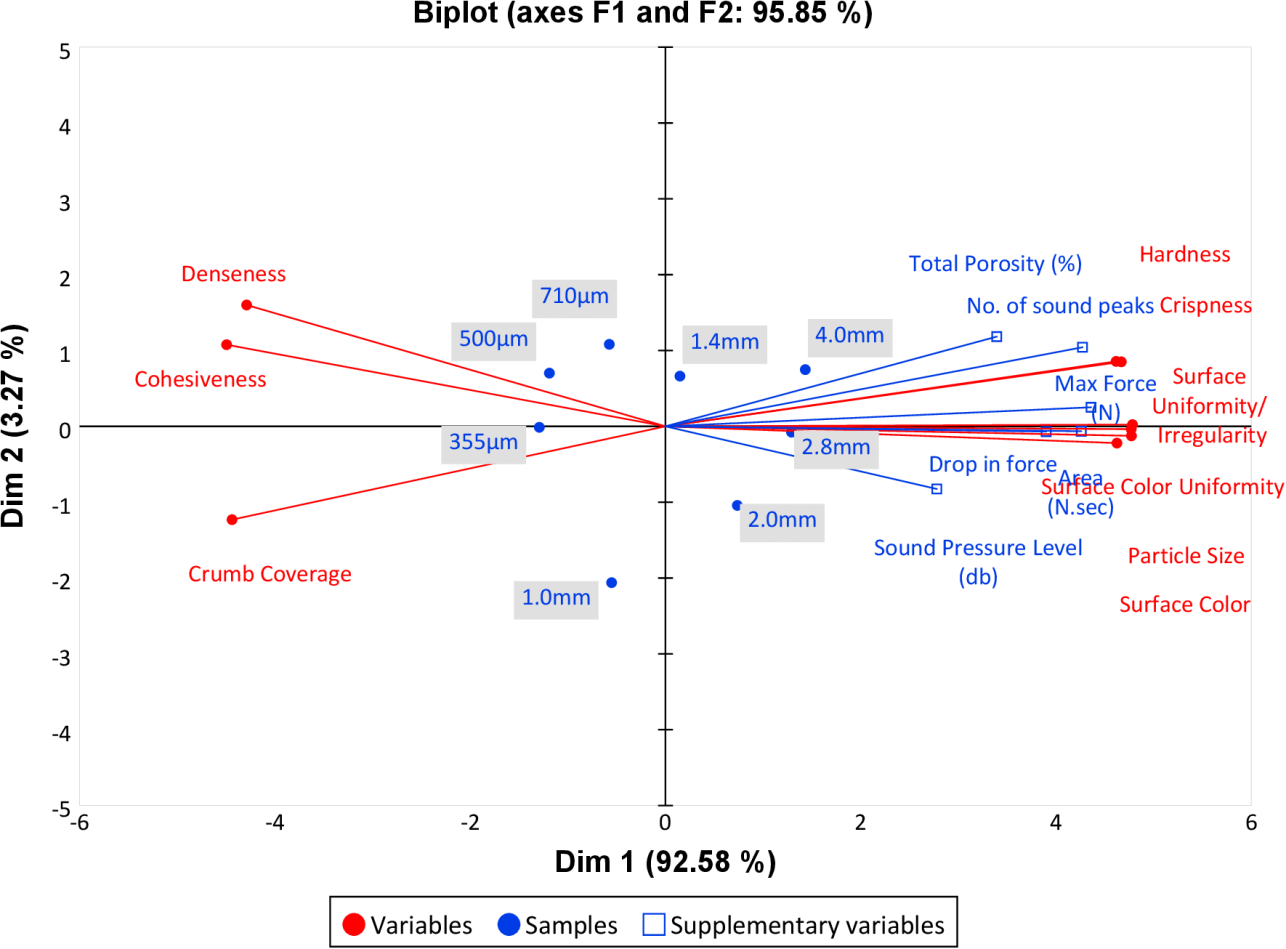
PCA biplot of first two principal components that explains 95.85% of variance for all sensory data and instrumental data. PCA, principal component analysis

Instrumental measurements of the same products were previously collected using X‐ray microCT, Texture Analyzer and Acoustic Envelope Detector (Voong et al., 2018). A combination of internal morphology characterization, force‐deformation, and acoustic emission have been used to characterize crispness instrumentally. Instrumental and sensory variables were studied together using PCA (Figure 2). Figure 2 displays samples 4.0, 2.8, 2.0, and 1.4 mm to be close in proximity, indicating similarities between all four. Figure 2 suggests 4.0, 2.8, 2.0, and 1.4 mm to score highly for total porosity, sound peaks, maximum force, drop in force, area, and sound pressure level. Samples 710, 500 and 355 μm are in close proximity to each other but are scoring low for all instrumental attributes.

Larger breadcrumbs are highly amorphous, therefore surface irregularity can be expected. Irregular structures will also have protrusions that are more exposed during frying. A higher exposure will lead to over cooking, therefore prolonging Maillard reactions will lead to browning. A prolonged oil and moisture exchange will also lead to deeper surface drying, therefore a harder and crispier crust. This explains the higher scores for “surface color”, “color uniformity”, “crispness” and “hardness”. The smaller breadcrumb coating sizes, such as 1.0 mm, 710 μm, 500 μm and 355 μm scored highly for cohesiveness, denseness and crumb coverage. A higher packing ability will provide a stronger crust barrier against oil penetration (Dana & Saguy, 2006), therefore more moisture is retained. This explains why ‘crispness’ and ‘hardness’ scored lower for the smaller breadcrumb coating sizes as oil and moisture has not occurred sufficiently enough to dry out the crust.

### 3.3 Clustering samples to highlight similarities

**Figure 3:**
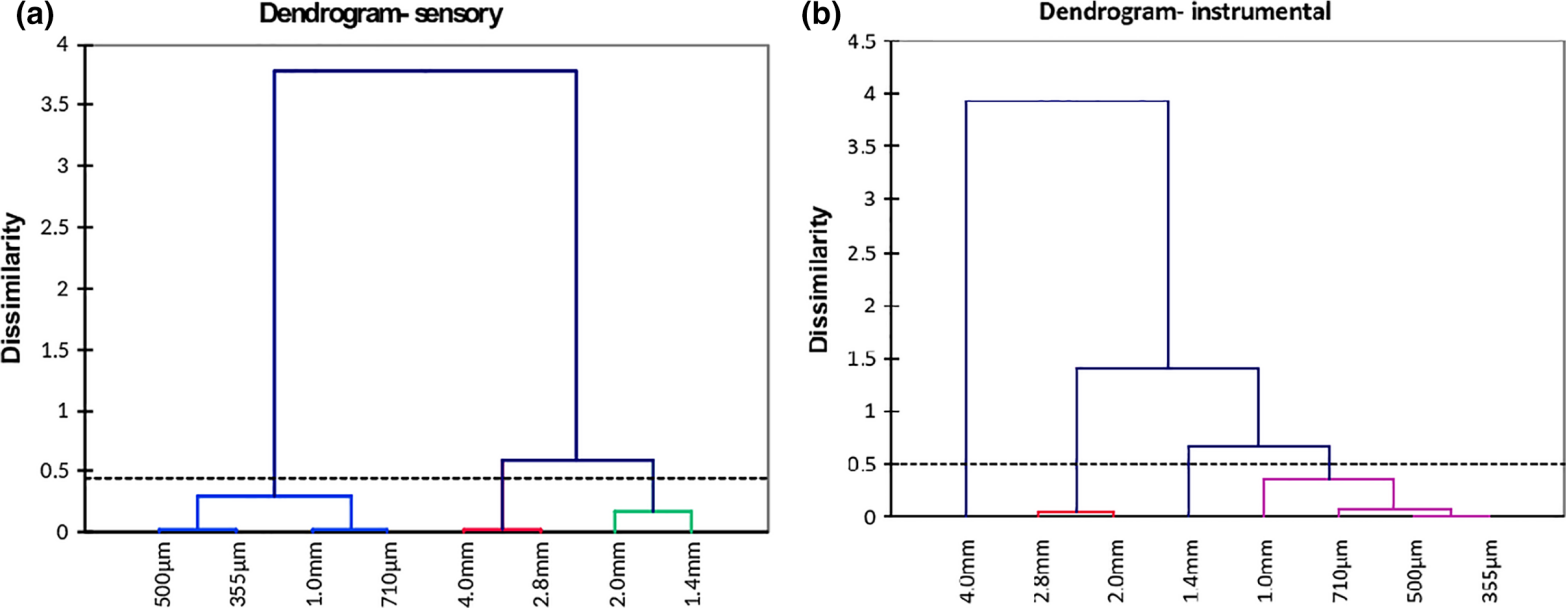
Dendrograms displaying the progressive clustering of samples when assessed by (a) sensory measurements and (b) instrumental measurements. Truncated lines indicate where classes have been defined by wards method

Agglomerative Hierarchical Clustering analysis is a classification method that investigates dissimilarities between observations (Qannari, Vigneau, Luscan, Lefebvre, & Vey, 1997). Figure 3 shows that different clusters were identified when evaluating samples by either sensory measurements or instrumental measurements. When evaluating samples with sensory measurements, clustering analysis results in three groups; 4.0 with 2.8 mm, 2.0 with 1.4 mm, and lastly 1.0 mm with 710, 500, and 355 μm. Instrumental measurements appear to find more clusters that is, more dissimilarities between samples. Four clusters were identified; 4.0 mm as its own cluster, 2.8 mm with 2.0 mm, 1.4 mm as its own cluster and 1.0 mm with 710, 500, and 355 μm.

Figure 3a clusters 4.0 mm with 2.8 mm, these samples are the largest breadcrumb sizes and score highly for all instrumental variables. This is further explained as 4.0 mm and 2.8 mm are not significantly different in terms of crispness (*p*‐value .999), hardness (*p*‐value .667), cohesiveness (*p‐*value .939) and denseness (*p*‐value 1.00). Surface color (*p*‐value 1.00), particle size (*p*‐value .321), surface irregularity (*p*‐value .865), crumb coverage (*p*‐value 1.00), and color uniformity (*p*‐value .999). Figure 3b clusters 4.0 mm in to its own cluster, indicating that more differences are found instrumentally than via sensory testing.

Sensory cluster of 2.0 and 1.4 mm are not significantly different in terms of crispness (*p*‐value 1.00), hardness (*p*‐value 1.00), cohesiveness (*p*‐value .646), denseness (*p*‐value .841), surface irregularity (*p*‐value .275), and crumb coverage (*p*‐value .945).

Figure 3b shows 1.4 mm in its own cluster, this again indicates that instrumental measurements are more differentiating. Both sensory and instrumental dendrograms cluster 1.0 mm, 710 μm, 500 μm, and 355 μm, all of which are the smaller breadcrumb sizes. Table 2 shows these samples are not significantly different in terms of crumb coverage, denseness or hardness.

The composition in both dendrograms are in fact similar, both show that samples are clustering in order of breadcrumb size.

### 3.4 Instrumental and sensory data relationships

**Figure 4:**
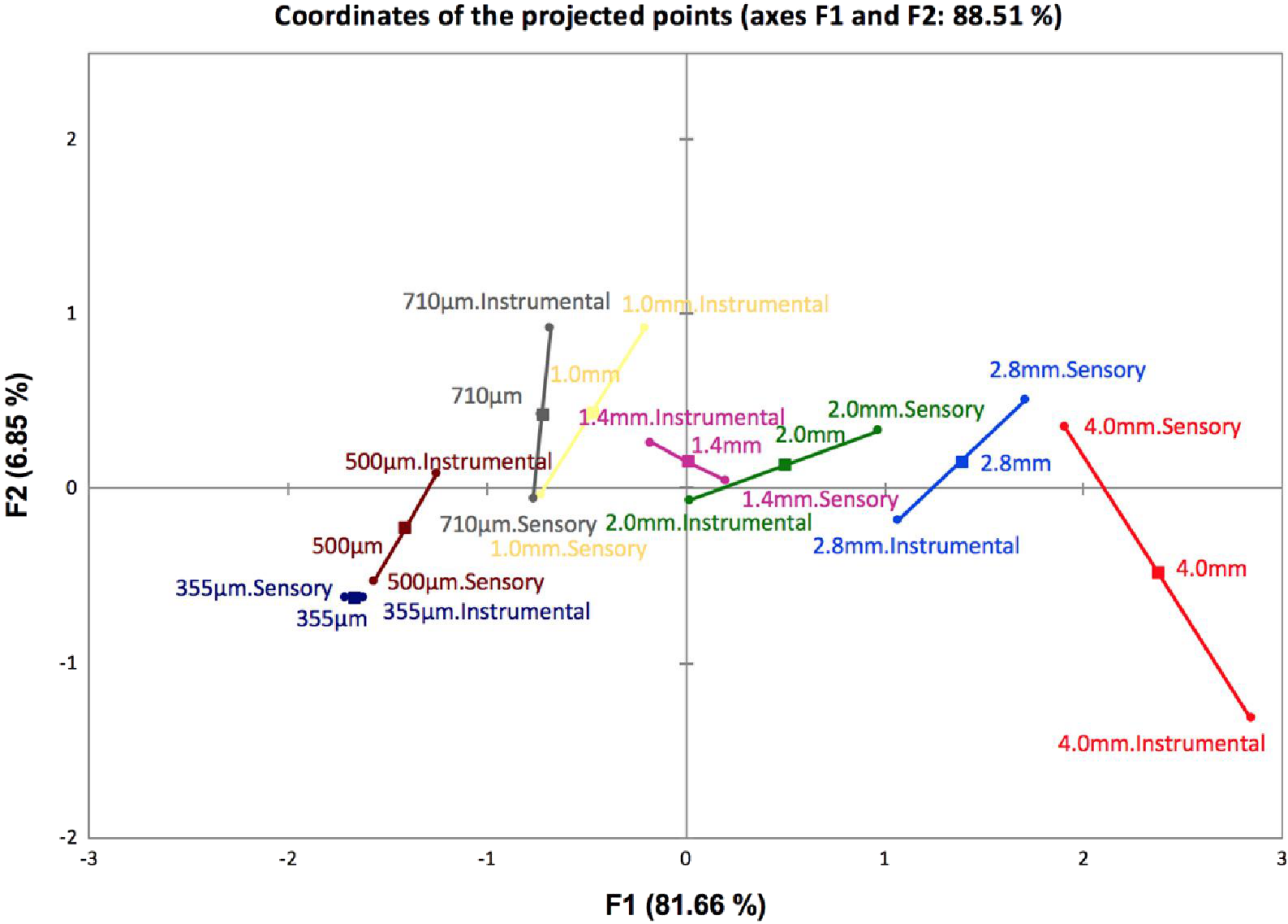
Two‐dimension MFA plot illustrating the correlation of instrumental and sensory measurements of crispness for each sample. MFA, multiple factor analysis

Sensory and instrumental data were analyzed jointly using MFA to study relationships between instrumental and sensory measurements. As shown in Figure 4, the first two dimensions explain a total variance of 88.51%. Each sample is represented by a central point, two partial points are then projected away from the central point. Each projected end representing either sensory measurements or instrumental measurements. If the two projected points are in close proximity to one another, this indicates agreement between sensory and instrumental measurements. Alternatively, if the two projected points are a part, this indicates discordance (Pagés & Husson, 2005). Figure 4 shows that sample 355 μm to have the greatest agreement whilst sample 4.0 mm has the greatest discordance between it's sensory and instrumental data. In general, as breadcrumb size decreases, agreement between sensory and instrumental increases. This can be explained as coatings with large breadcrumbs are amorphous in size, shape and therefore surface coverage. Subsequently, this will affect its mechanical and physical properties upon frying as well as sensory perception. Therefore, higher variability can be expected from instrumental and sensory measurements. This is further supported by Figure 4, as the position of the instrumental point of sample 4.0 mm is the furthest point from all others, suggesting that the instrumental measurements for sample 4.0 mm are more different than the other samples.

The length and orientation of the line provides an indication of how well sensory and instrumental measurements correlate (Cadena et al., 2013). The orientation of the lines suggests whether samples are similar or not for example, samples 4.0 and 2.8 mm are more similar in terms of sensory measurements than instrumental measurements. Figure 4 also highlights the composition of the samples, from left quadrants to right quadrants, they are arranged according to breadcrumb size.

The RV coefficient can be used to assess the global similarity between two matrices (Cadena et al., 2013). An overall *RV* value of 0.810 suggests that instrumental and sensory data are highly correlated. Table 4 highlights that all X‐ray microCT, texture analysis and acoustics correlate highly with sensory texture and appearance attributes. MFA results demonstrate how a combination of both types of testing allow for a fundamental understanding of how physical parameters relate to sensory parameters. Crispness is ultimately a result from the structural properties of the food product (Mallikarjunan, 2004).

**Table 4:** MFA RV coefficients highlighting correlations between types of instrumental measurements to types of sensory measurements

	X‐ray microCT	Texture analyzer	Acoustic	Sensory texture	Sensory appearance	MFA
X‐ray microCT	1.000					
Texture analyzer	0.787	1.000				
Acoustic	0.614	0.618	1.000			
Sensory texture	0.484	0.788	0.727	1.000		
Sensory appearance	0.518	0.799	0.717	0.971	1.000	
MFA	0.779	0.913	0.846	0.909	0.917	1.000

Abbreviation: MFA, multiple factor analysis.

### 3.5 Predictive modeling of crispness using instrumental variables

Multiple linear regression was previously carried out to test for multicollinearity and to provide validation for using PLS regression. PLS was used with the aim to predict sensory crispness from instrumental parameters (Equation 1). Crispness was selected as the dependent variable as it can be considered to be one of the most important attributes when assessing the quality and freshness of deep‐fried battered and breaded goods (Mellema, 2003). Although, it can be noted that more than one dependent variable can be predicted using PLS regression (Meilgaard et al., 2016). The PLS correlation matrix shows that crispness to be one of the most highly correlated attribute with porosity (*r* = .740), maximum force (*r* = .882), drop in force (*r* = .865), sound peaks (*r* = .926) and sound pressure level (*r* = .618). This further supports the relationship of instrumental measurements to sensory perception of crispness. The advantage of this predictive model means that mechanical and physical properties can be used to predict a textural property. Therefore, a sensory panel is not required and less input variables are needed for the simulation of the model. This is potentially less time‐consuming and more cost‐effective.

Crispness=2.45+9.00E−03×Total porosity+0.0140×Maximum force+4.82E−03×Area+0.614×Drop in force+1.038E−02×Number of sound peaks+5.16E−02×Sound pressure level

$$R^{2}=.854$$

**Figure 5:**
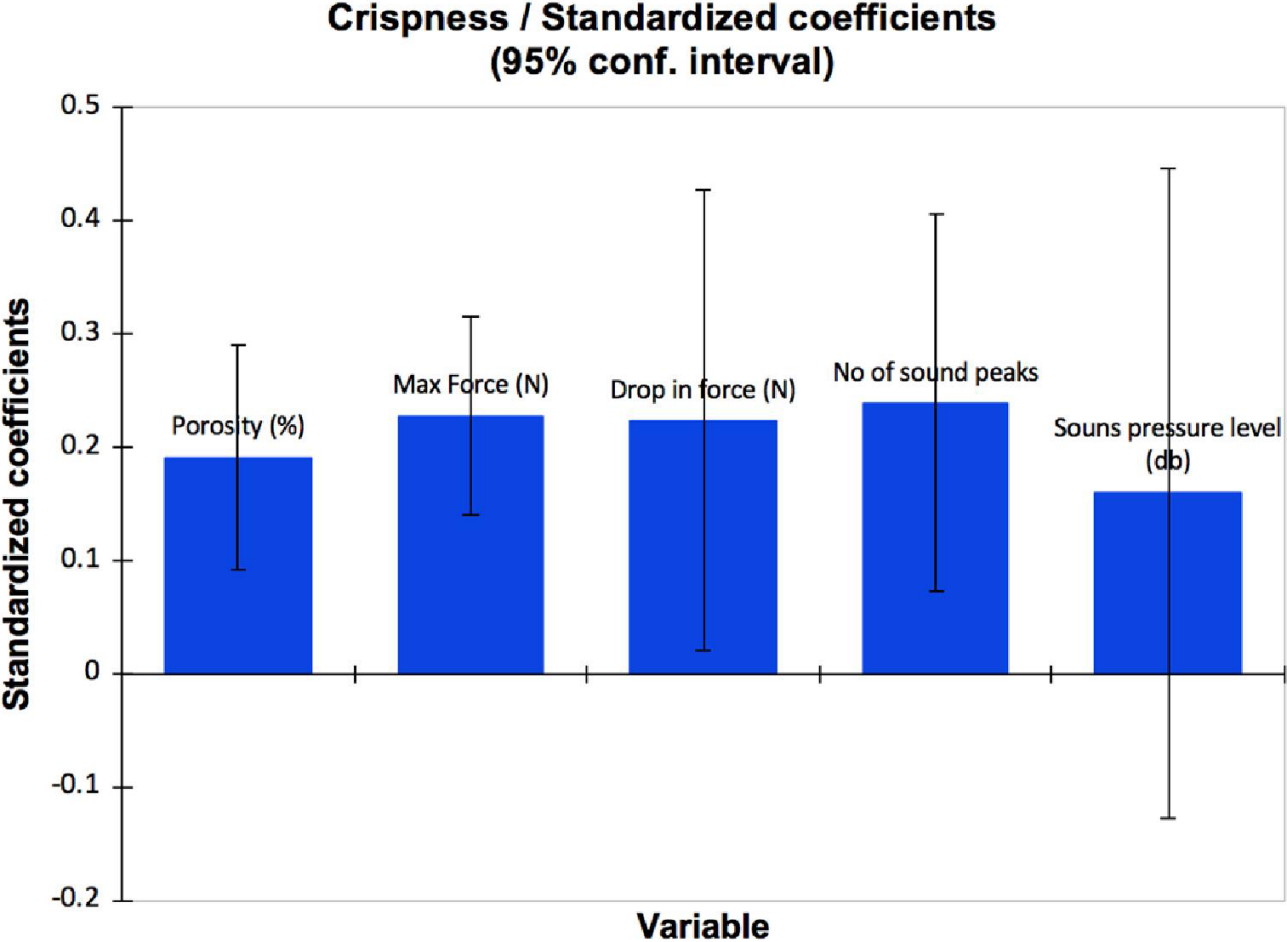
The standard coefficients of instrumental model associated to each dependent variable. Total porosity was collected from X‐ray microCT. Maximum force and drop in force was collected from texture analyzer. Number of sound peaks and sound pressure level was collected from an acoustic envelope detector

**Figure 6:**
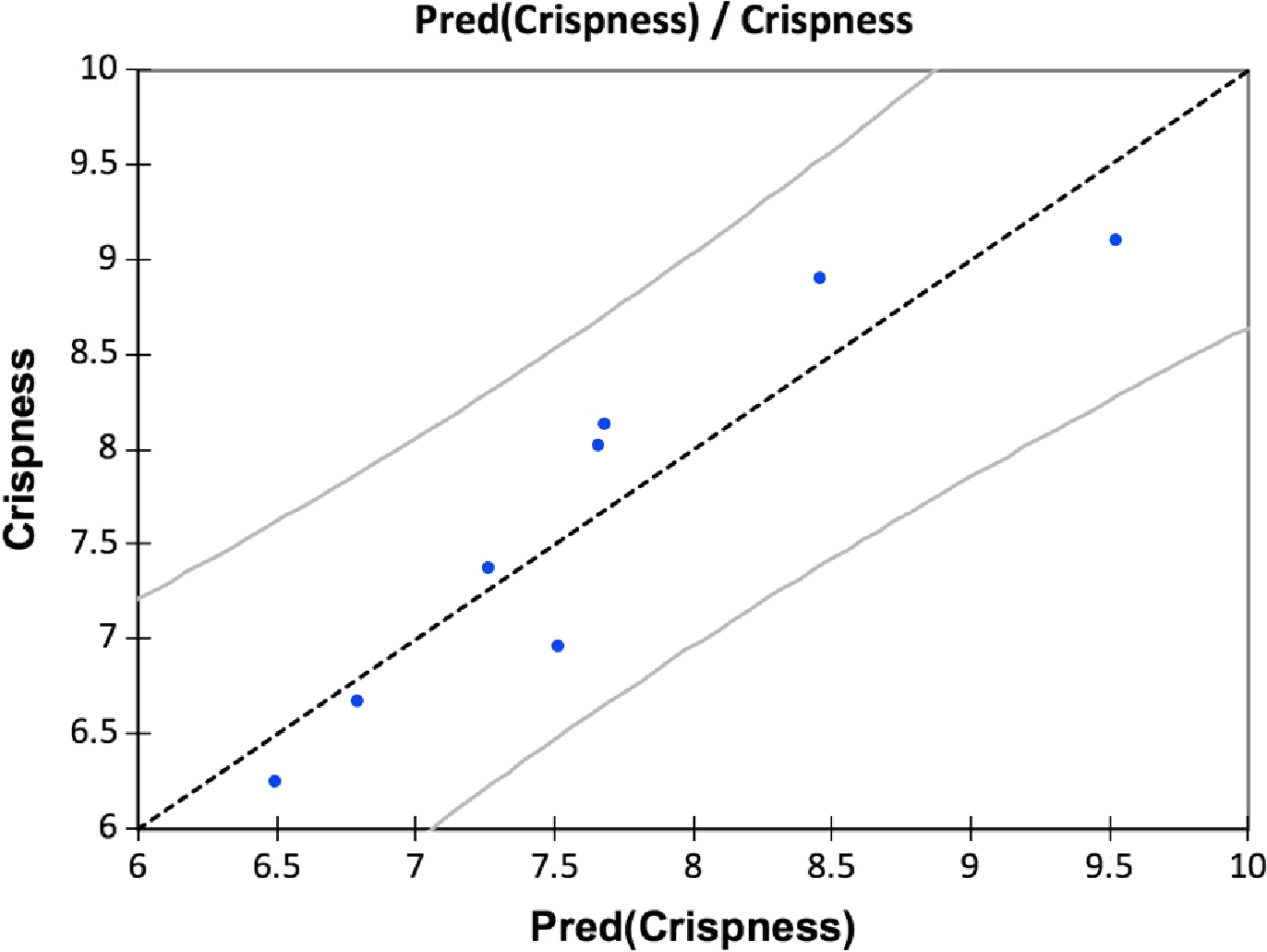
Residuals of predicted values of crispness vs observations for Equation 1 showing predictability of model

A *R* ^2^ value of .854 indicates high predictive ability of this model (Henseler, Ringle, & Sinkovics, 2009). This is also supported by a *Q* ^2^cum value of 0.770, which also measure the goodness of fit and predictive quality. Sensory crispness of deep‐fried battered and breaded shrimps could be predicted by resimulation of this model. As shown in Figure 5, all instrumental variables are positively correlated with the predictive ability of Equation 1, the larger the column indicates a larger influence on the model. Figure 6 displays the predictive quality of the model with residual plots, all points lie within threshold (gray lines), this indicates high predictive ability.

PLS regression has been previously used to correlate instrumental data and sensory data for meats (Castejón, García‐Segura, Escudero, Herrera, & Cambero, 2015), fruits (Chang, Vickers, & Tong, 2018), dairy (Shiota, Iwasawa, Suzuki‐Iwashima, & Iida, 2015) and dry snacks (Moghaddam, Razavi, Taghizadeh, & Sazgarnia, 2016). However, the use of PLS to predict crispness in deep‐fried battered and breaded goods is limited.

## 4 CONCLUSION

In conclusion, the main contributors of crispness in deep‐fried battered and breaded coatings have been investigated in this study. In terms of sensory attributes, PCA showed that crispness was highly correlated with hardness, particle size, surface color, number of sound peaks, color uniformity, and surface uniformity, this was dependant on breadcrumb size.

MFA confirmed positive correlations between instrumental and sensory measurements, this was also dependant on breadcrumb size. AHC identified that coatings were clustering into three groups; large, intermediate and small breadcrumb size. These clusters share common sensory and instrumental attributes within their samples. Crispness was found to be one of the most highly correlated variables to instrumental data, this allowed a predictive model to be developed using PLS regression analysis. An instrumental predictive model that allows prediction of crispness will allow a degree of quality control in future products. The use of force‐deformation coupled with acoustic events as well as internal morphology characterization allows for a thorough understanding of how the microstructure relates to texture perception.

## ETHICAL STATEMENTS

Conflict of Interest: All authors of this manuscript declare that they do not have any conflict of interest.

Ethical Review: This study was approved from the ethics committee at the University of Birmingham.

Informed Consent: All participants involved in the sensory study gave written informed consent prior to beginning this study.

Author Contributions: Author K.Y.V. constructed the manuscript, collected and analyzed the data. Authors A.N.W, T.B.M., I.T.N. supervised the project and proof‐read the manuscript.
